# Supplementary material for: Bacterial RadA is a DnaB-type helicase interacting with RecA to promote bidirectional D-loop extension
Source: Nat Commun. 2017 May 31;8:15638. doi: 10.1038/ncomms15638 (PMC5512693; doi:10.1038/ncomms15638)
Supplement: Supplementary Information — Supplementary Figures and Supplementary Table [file ncomms15638-s1.pdf]

a

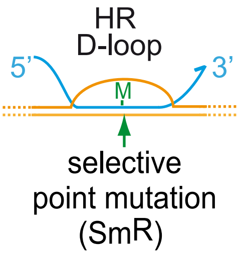

	wt	*radA*⁻	C27A	K101A	K251A	R253A
**relative transformation efficiency**	1	0.009	0.012	0.070	0.018	0.008
**SD**	-	0.006	0.005	0.020	0.002	0.008

e

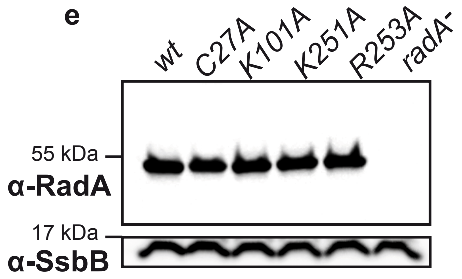

b

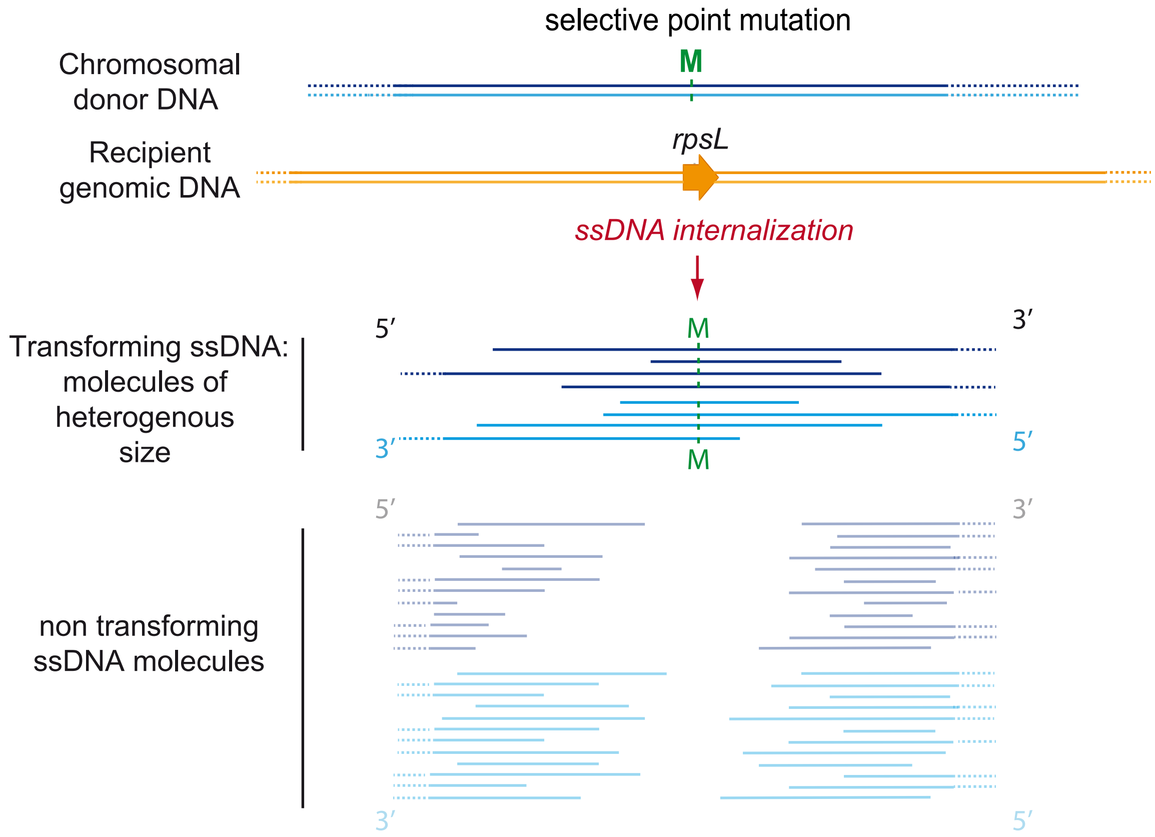

c

PCRc ($ng.mL^{-1}$)	300	30	3
% of transformation (SmR)			
wild-type strain	87.5	66	18.6
radA⁻ strain	3.5	1	0.13
transformation deficit of *radA* mutant			
	**25**	**66**	**143**

d

Donor PCR molecules	PCRc	PCRe	
% of transformation (NovR)			
wild-type strain	88	31	(2.8)
radA⁻ strain	5.7	0.06	(95)
transformation deficit of *radA* mutant			
	15	516	

PCRc	PCRe	
% of transformation (RifR)		
100	35	(2.8)
3.8	0.028	(136)
transformation deficit of *radA* mutant		
28	1250	

Supplementary Figure 1. Effect of RadA deletion and of point mutations in the C4 and H domains on pneumococcal transformation.

(**a**) Relative transformation efficiencies of *radA* point mutants compared to wild type (normalized to 1) were measured using chromosomal donor DNA carrying a streptomycin resistance point mutation (Sm^R) in the *rpsL* gene. SD: standard deviation of the mean calculated from triplicate repeats. (**b**) Schematic representation of transforming (bold) and non-transforming ssDNA fragments internalized during transformation with chromosomal DNA: a large majority of ssDNA fragments internalized by competent cells are non-transforming ssDNA; transforming ssDNA molecules arising from the two strands, depicted in dark and light blue, are of undefined size and the position of the mutation on these fragments is random. The transformation frequency can reach 10% with a saturating amount of chromosomal donor molecules and 100% with PCR fragments (panel d, below), indicating that all cells in the population can be transformed. In the absence of RadA, the transformation frequency drops to 0.1% with chromosomal donor DNA, showing that RadA helps the incorporation of unbiased transforming ssDNA molecules. (**c**) Transformation frequencies obtained for *radA*$^-$ (R2194) and its parental strain (R1818) with various concentrations of PCRc carrying a point mutation conferring Sm^R. See also Figure 1. (**d**) Transformation frequencies obtained for *radA*$^-$ (R2194) and of its parental strain (R1818) with PCRc or PCRe products (30 $ng.ml^{-1}$) homologous to the *gyrB* locus and carrying a point mutation conferring novobiocin resistance (Nov^R) or to the *rpoB* locus and carrying a point mutation conferring rifampicin resistance (Rif^R). The numbers in parenthesis refer to the relative deficit of transformation between PCRc and PCRe donors for the same strain. Transformation frequencies were measured in three independent experiments, leading to similar relative differences between PCRe and PCRc fragment transformation efficiency. (**e**) Western blot analysis of total cell extracts prepared from pneumococcal wild type and *radA* competent cells, using antibodies raised against pneumococcal RadA. The competence-induced SsbB protein was detected as control using antibodies raised against SsbB.

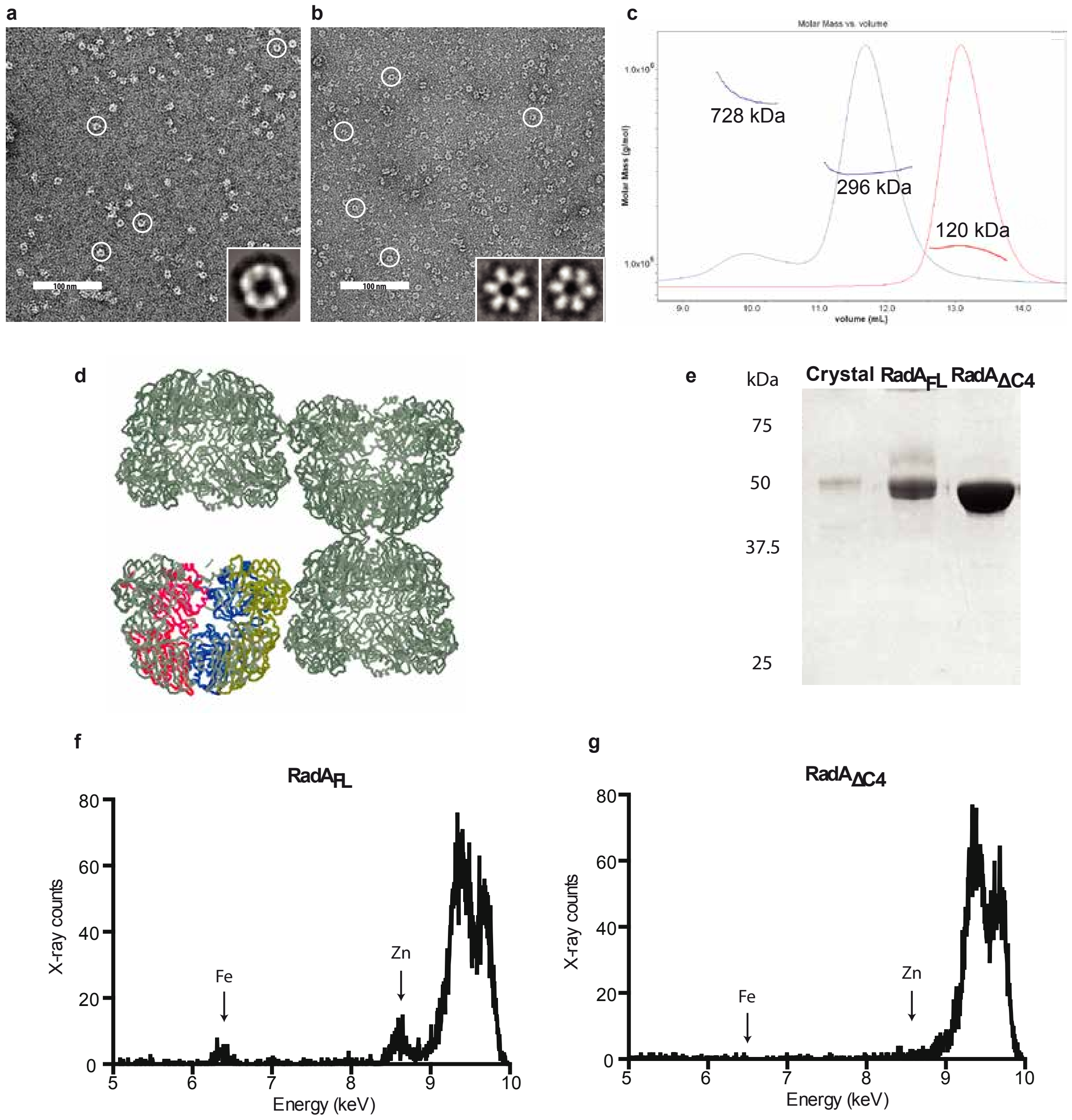

Supplementary Figure 2. Pneumococcal RadA forms hexamers and multimers in solution and has a functional Zinc finger.

(**a-b**) Negatively stained EM images of RadA$_{FL}$ (**a**) and of RadA$_{P}$ (**b**). (**a**) The inset shows the 2D class average of 340 particles RadA$_{FL}$ self-assembled into ring-shaped hexamers. (**b**) RadA$_{P}$ self-assembles into ring-shaped hexamers. The 2D averages show two possible conformations of the RadA$_{P}$ hexamer: open (left, n=5) and closed (right, n=50), the latter being the most

abundant species observed on the grid. (**c**) MALS/QELS/UV/RI analysis of $RadA_{FL}$ (blue) and of the $RadA_P$ domain (red), with the respective molecular weights calculated using ASTRA software. The predicted molecular weights of the hexamers of $RadA_{FL}$ and $RadA_P$ are 295.8 and 127.7 kDa respectively. (**d**) Crystal packing of $RadA_{FL}$. A large gap is present between hexamers in the crystal packing of $RadA_{FL}$.(**e**) $RadA_{FL}$ crystal content analysis. 12% SDS gel electrophoresis of a dissolved crystal of $RadA_{FL}$ (Crystal), and of the $RadA_{FL}$ and $RadA_{\Delta C4}$ protein samples prior crystallization. (**f-g**) XRF spectra of $RadA_{FL}$ (f) and $RadA_{\Delta C4}$ (g), reveal the presence of Zn and Fe only in $RadA_{FL}$, demonstrating that the C4 domain of RadA is a zinc-binding motif and that the C4 domain is present but not ordered in the $RadA_{FL}$ crystal.

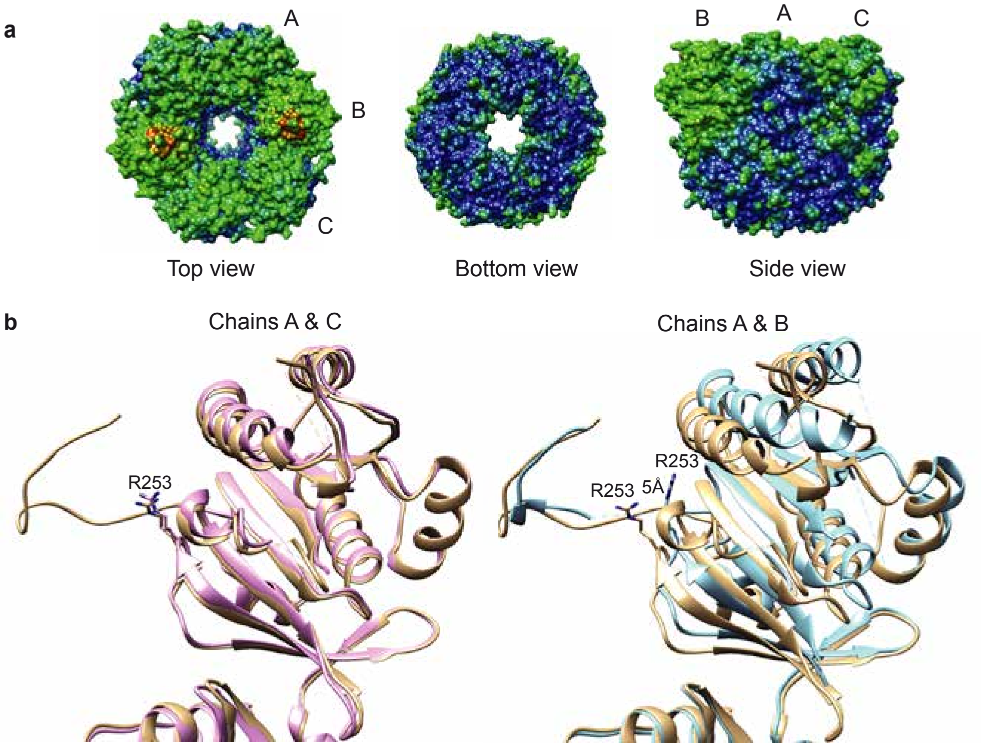

Supplementary Figure 3. The H domain of RadA is flexible.

(**a**) B factor distribution in the $RadA_{FL}$ hexamer. B factors reflect structural heterogeneity and disorder. The $RadA_{P}$ domain of all protomers in the hexamer has the lowest B factor values, ranging from 56 to 217 (blue to green). The H and C4 domains have higher B factors, ranging from 217 to 378 (green to red). In particular, chain B of the trimeric unit of $RadA_{FL}$ in the crystal appears to have the highest B factor and thus contains the most residues of uncertain position.

(**b**) The RadA H domain overlaps perfectly with the three monomers of the trimeric unit. When the RadA H domains are aligned, those of chains A (taupe) and C (pink) overlap well (RMSD between 372 atom pairs is 0.831 Å), whereas those of chains A (taupe) and B (blue) are shifted by 5 Å (RMSD between 208 atom pairs is 0.739 Å).

a

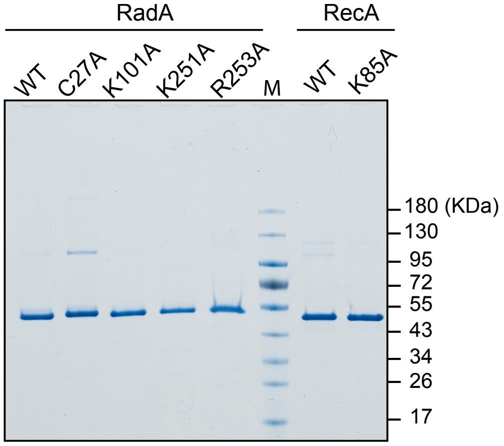

b

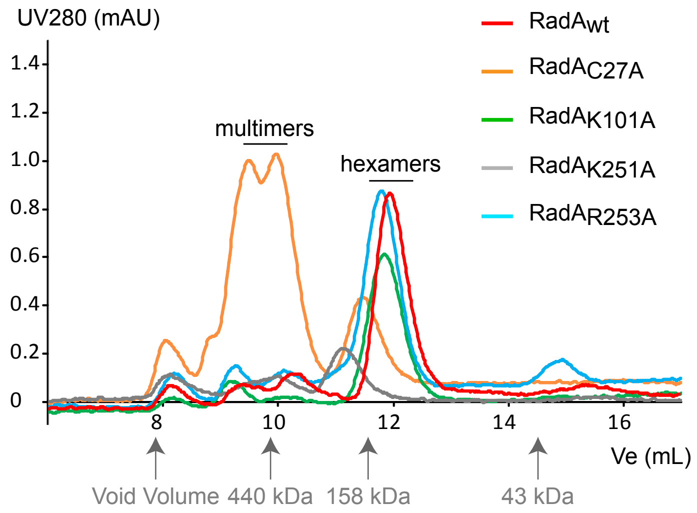

c

	RadAwt + ssDNA	RadAwt + dsDNA	RadAwt no DNA	RadAC27A + ssDNA	RadAK101A + ssDNA	RadAK251A + ssDNA	RadAR253A + ssDNA
Best-fit and Standard Error							
Vmax	4.79 (0.19)	0.99 (0.05)	0.1 (0.01)	6.48 (0.33)	0.75 (0.04)	0.24 (0.03)	N.D *
Km	0.15 (0.03)	0.19 (0.04)	0.06 (0.06)	0.22 (0.05)	0.35 (0.06)	0.32 (0.15)	N.D *

d

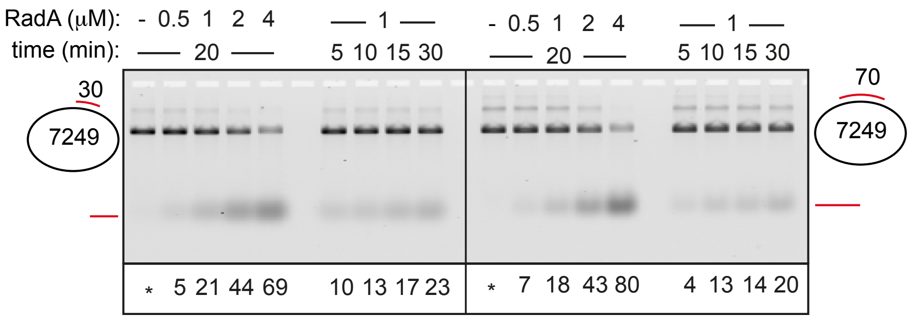

Supplementary Figure 4. Purification of RadA and RecA derivatives and ATPase activity of RadA proteins.

(**a**) Purified RadA and RecA proteins subjected to SDS-PAGE and revealed by Coomassie blue staining. A minor species in the $RadA_{C27A}$ preparation migrates at the position expected for a dimer of RadA; the tendency of this RadA mutant to self-assemble in the form of multimers (see also b) is consistent with this band being a dimer form. (**b**) Gel filtration analysis of purified RadA on a Superdex200 analytical sizing column. The calibration of the column performed with purified protein standards is indicated as grey numbers below the elution chromatogram. (**c**) ATPase activity of RadA derivatives. V_{max} and K_m values were calculated from the data of Figure 4 using Michaelis-Menten analysis and best-fit. Standard error values were calculated using GraphPad-Prism 7 software from the data of three independent experiments. N.D *: not determined, since the ATPase activity of $RadA_{R253A}$ was too weak to fit a Michaelis-Menten curve. (**d**) Helicase assays performed on M13mp18 ssDNA hybridized either with a Cy3-fluorescent (red) 30-mer (left panel) or 70-mer (right panel). Both DNA substrates were submitted in parallel to increasing amount of RadA (0.5 to 4 μM) for 20 min or to a fixed RadA concentration (1 μM) for 5 to 30 min. Values at the bottom of each lane correspond to the relative % of unwound Cy3-oligonucleotide (detailed in methods). (*) corresponds to the reference lane, without protein .

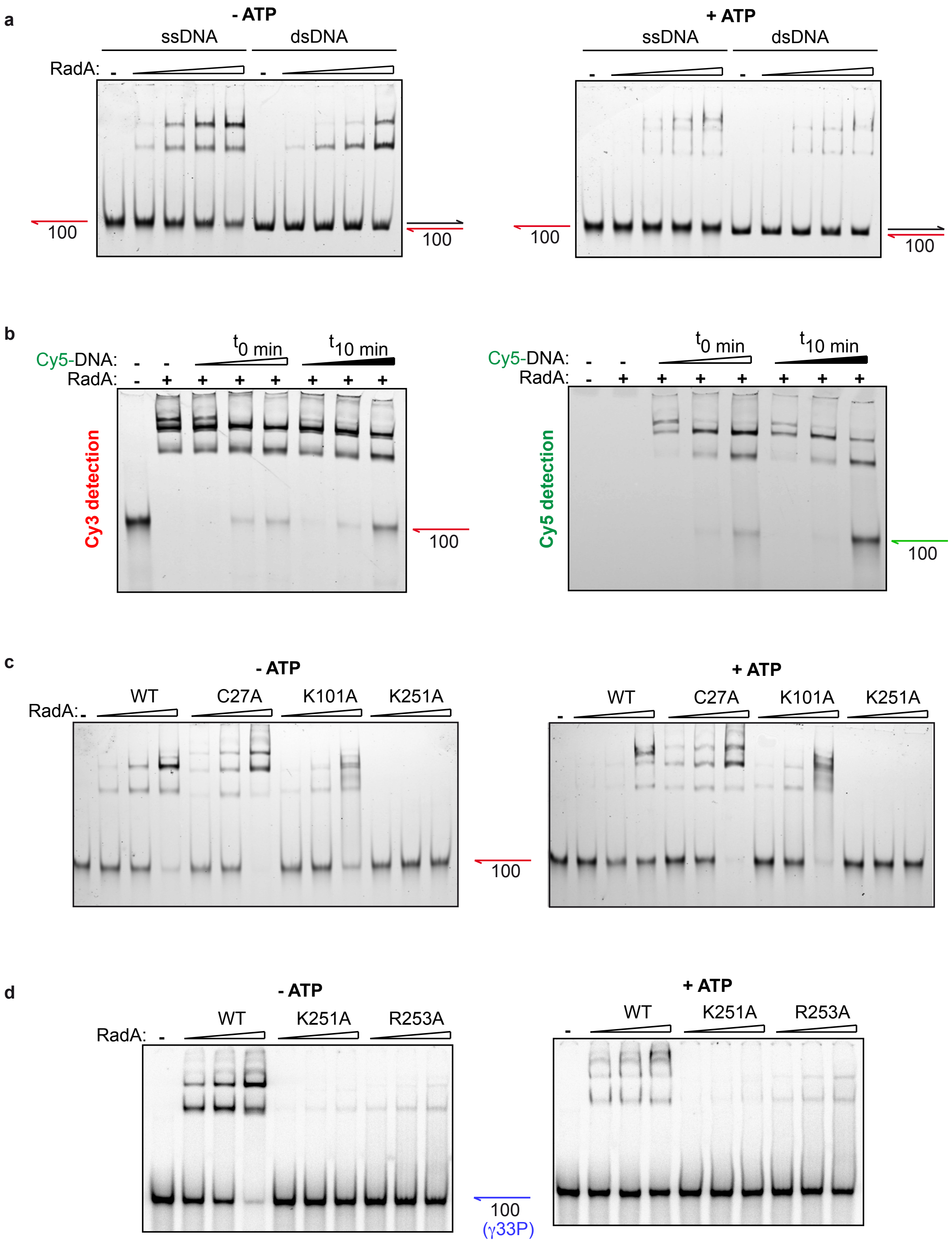

a
- ATP
ssDNA
dsDNA
RadA:
100
+ ATP
ssDNA
dsDNA
100
b
Cy5-DNA:
RadA:
t0 min
t10 min
Cy3 detection
100
Cy5-DNA:
RadA:
t0 min
t10 min
Cy5 detection
100
c
- ATP
RadA:
WT
C27A
K101A
K251A
100
+ ATP
WT
C27A
K101A
K251A
d
- ATP
RadA:
WT
K251A
R253A
100
(γ33P)
+ ATP
WT
K251A
R253A

Supplementary Figure 5. Electrophoretic mobility Shift Assay (EMSA) of ssDNA and dsDNA binding by RadA derivatives.

(**a**) EMSA performed with 50, 250, 500 and 750 nM of RadA and 100 nt long Cy3-labelled ssDNA or dsDNA, in the absence (left) or presence (right) of ATP. (**b**) EMSA performed with 750 nM of RadA and 10 nM Cy3-labelled ssDNA (100 nt) in the presence of 5, 10 or 20 nM of Cy5-labelled ssDNA (100 nt) added either at the same time (t_0 min) or after 10 min of incubation (t_{10} min) followed by further incubation for 10 min. The same gel was used to detect Cy3- (left) or Cy5- (right) specific fluorescent signals. The patterns observed by adding increasing amounts of Cy5-ssDNA to RadA pre-bound or not to Cy3-ssDNA are nearly identical. This shows that RadA interaction with the two ssDNA probes equilibrates between them rapidly. (**c**) EMSA performed with 80, 250 and 750 nM of wild-type and mutant RadA proteins in the absence (left) or presence (right) of ATP, with Cy3-ssDNA (100 nt). (**d**) As in (c), but with ^{33}P-radio-labelled ssDNA instead of Cy3 ssDNA, to investigate ssDNA binding of wild-type RadA in comparison with $RadA_{K251A}$ and $RadA_{R253A}$ mutants (150, 300 and 600 nM). In this assay, which provides a more sensitive detection of labeled ssDNA, $RadA_{K251A}$ and $RadA_{R253A}$ mutants are seen to be strongly affected but not completely defective in ssDNA binding. See also Figure 4.

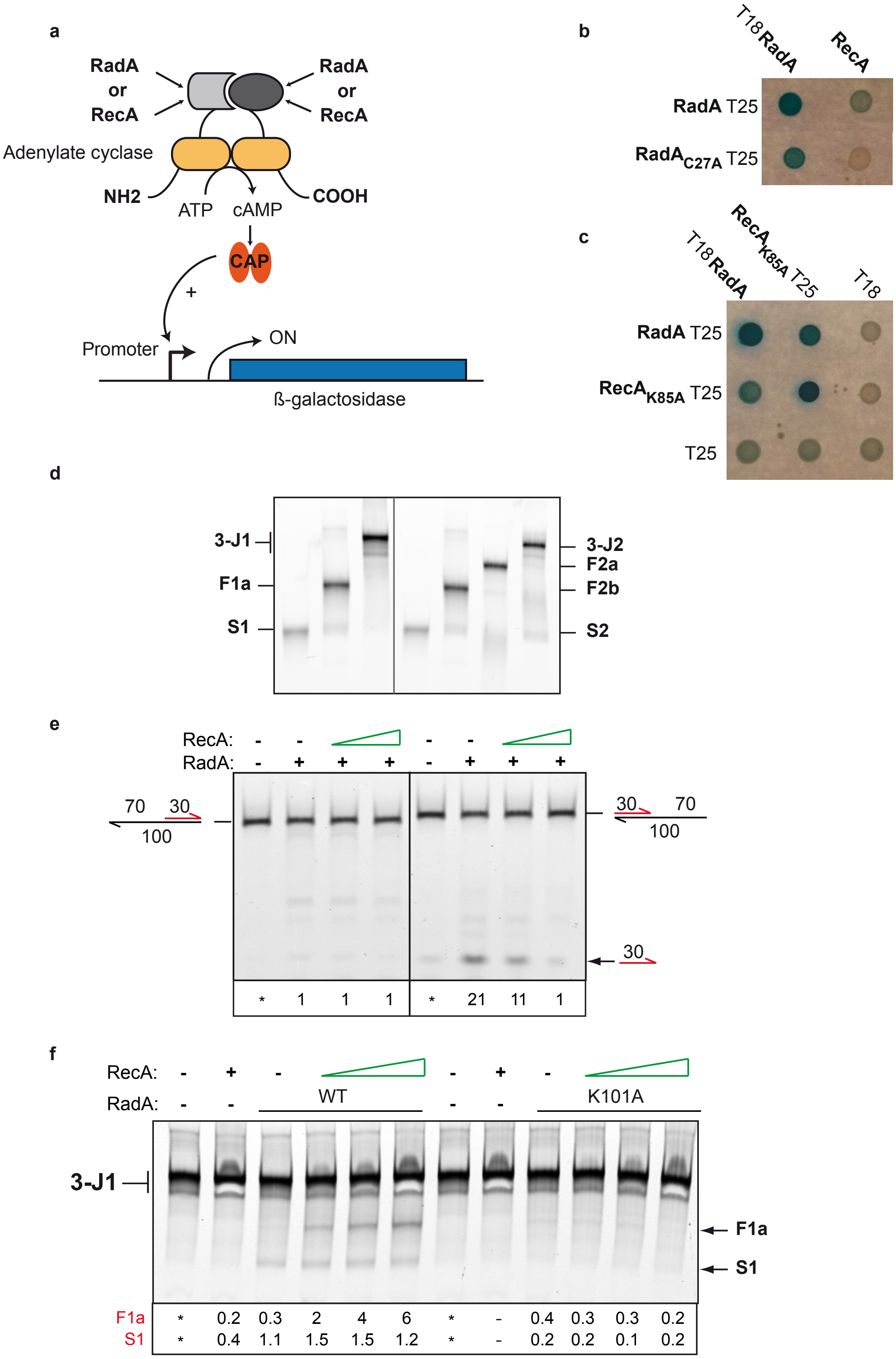

a
RadA
or
RecA
RadA
or
RecA
Adenylate cyclase
NH2
ATP
cAMP
COOH
CAP
+
Promoter
ON
ß-galactosidase
b
T18 RadA
RecA
RadA T25
RadA C27A T25
c
T18 RadA
RecA K85A T25
T18
RadA T25
RecA K85A T25
T25
d
3-J1
F1a
S1
3-J2
F2a
F2b
S2
e
RecA:
RadA:
70
30
100
30
70
100
30
* 1 1 1 * 21 11 1
f
RecA:
RadA:
WT
K101A
3-J1
F1a
S1
F1a * 0.2 0.3 2 4 6 * - 0.4 0.3 0.3 0.2
S1 * 0.4 1.1 1.5 1.5 1.2 * - 0.2 0.2 0.1 0.2

Supplementary Figure 6. Interplay between RadA and RecA.

(**a**) Scheme of the BacTH assay. BTH101 *E. coli* cells, lacking endogenous ß-galactosidase and expressing RadA and RecA fused to either the T18 or T25 domain of Bordetella adenylate cyclase, were tested for ß-galactosidase expression on plates supplemented with IPTG and the chromogenic substrate X-Gal. If able to interact, the pair of fusion proteins tested reconstitutes adenylate cyclase to generate cAMP, which then activates the cAMP-bound catabolite activator protein (CAP) to induce the expression of ß-galactosidase. A positive interaction between two fusion proteins is scored by the appearance of blue color in the colony due to the breakdown of X-gal in the medium. (**b-c**) BacTH assay of with wild-type or mutated RadA and wild-type RecA (b) or with wild-type RadA and the $RecA_{K85A}$ mutant (c). See also Figure 5. (**d**) Gel migration profiles of 3-J and derivatives. (**e**) Helicase assays of RadA (750 nM) and with increasing amounts of RecA (300 and 600 nM) on a 3' (left panel) or 5' (right panel) tailed duplex Cy3-labeled on one strand (depicted in red; Supplementary Fig.8).

(**f**) Helicase assay performed with 750 nM of wild-type RadA and $RadA_{K101A}$ mutant proteins on 3-J1, in presence of 150, 300 and 600 nM RecA and ATP. Values at the bottom of each lane in (e-f) correspond to the relative % of unwound Cy3-oligonucleotide (detailed in methods). (*) corresponds to the reference lane, without protein.

a

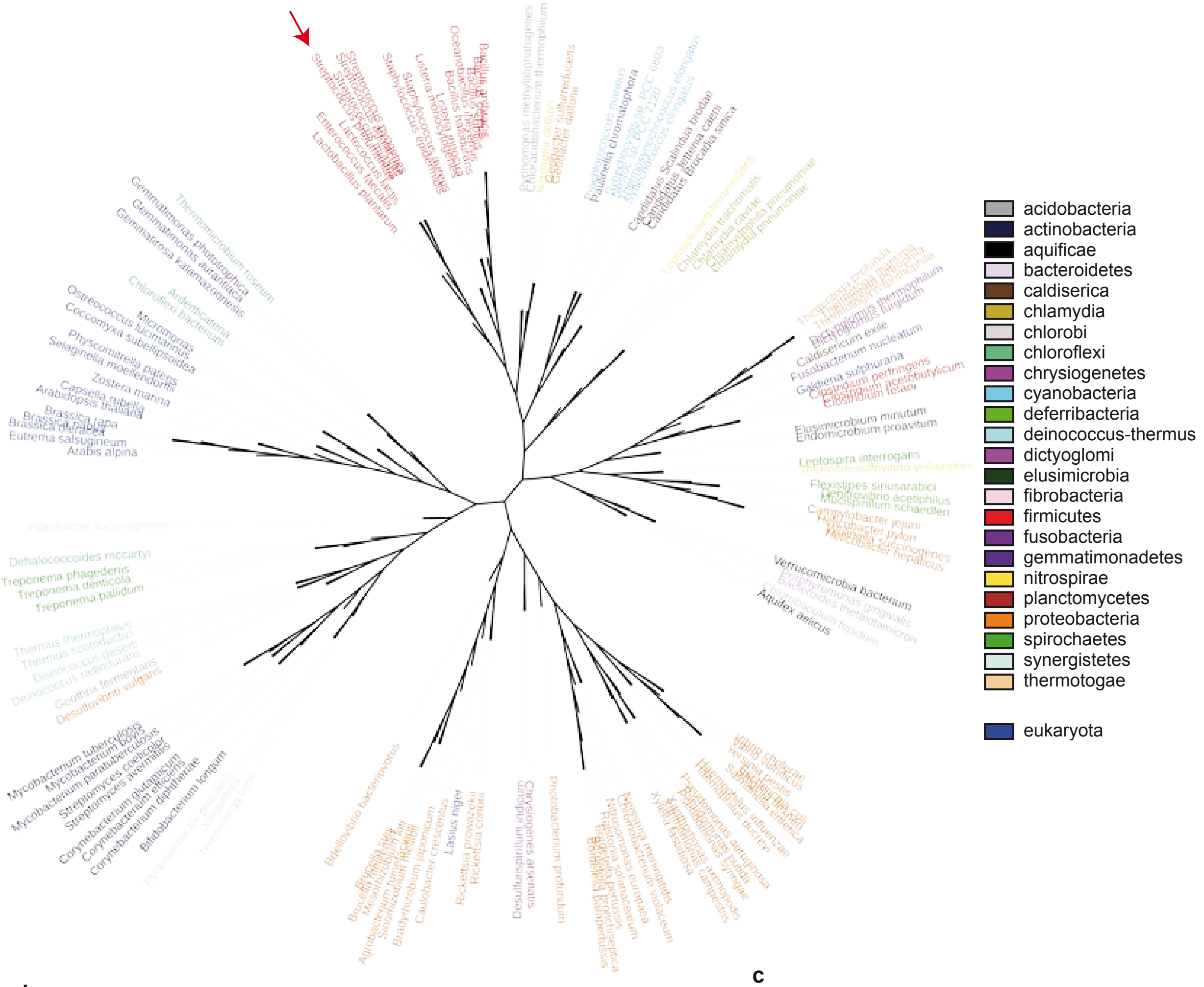

b

```
                                              H1  walker A               H1a
DnaB E. coli        209 TGYDDLNK-KTAGLQPSDLIIVAARPSMGKTTFAMNLVENAAMLQDKPVLIFSLEM----
Gp4  T7             290 SGCTGIND-KTLGARGGEVIMVTSGSGMGKSTFVRQQALQWGTAMGKKVGLAMLEESVEE
RadA S. pneumoniae  72  TEMEEFNRVLGGGVVPGSLVLIGGDPGIGKSTLLLQVSTQLSQVG--TVLYVSGEE----
                        :    :*     *    ..:::: .  .:**:*:  :   :  .        *     *
RadA consensus                           sviligGdPGiGKS                     vlYvsgEE

DnaB E. coli             PSEQIM-MRSLASLS---RVDQTKIRTGQLDDEDWARISGTMGILLEKRNIYIDDSSGLT
Gp4  T7                  TAEDLIGLHNRVRLRQSDSLKREIIENGKFD--QWFD------ELFGNDTFHLYDSF--A
RadA S. pneumoniae       SAQQIK---------------LRAERLGDIDS-----------------EFYLYAETNM-
                          ::::                      . *.:*                 :::   .
RadA consensus

                                              H2  walker B                         H3
DnaB E. coli             PTEVRSRARRIAREHGG--IGLIMIDYLQLMRVPALSDN--RTLEIAEISRSLKALAKEL
Gp4  T7                  EAETDRLLAKLAYMRSGLGCDVIILDHISIVVSASGESD--ERKMIDNLMTKLKGFAKST
RadA S. pneumoniae       ----QSVRAE----VERIQPDFLIIDSIQTIMSPEISGVQGSVSQVREVTAELMQLAKTN
                                   .           .:::* :. :       .     : ::   .*  :**
RadA consensus                                 lmviDSi                 vre-aa-lirlaK--

                                                         H4
DnaB E. coli             NVPVVALSQLNRSLEQR--ADKRPV-NSDLRESGSIEQDADLIMFIYRDEVYHENSDLKG
Gp4  T7                  GVVLVVICHLKNPDKGKAHEEGRPVSITDLRGSGALRQLSDTIIALERNQQ----GDMPN
RadA S. pneumoniae       NIAIFIVGHVTKE---------------GTLAGPRMLEHMVDTVLYFEGERH-------HT
                          : :. : :::..                *     :.:   * ::  :   :.
RadA consensus           gvavllvghv                  g-iAGPrvleHmvDtvl

DnaB E. coli              IAEIIIGKQR---------------NGPIGTV----------RLTFNGQWSRFDNYAGPQ 467
Gp4  T7                   LVLVRILKCRFTGDTGIAGYMEYNK--ETGWLEPSSYSGEEESHSESTDWSNDTDF---- 566
RadA S. pneumoniae        FRILRAVKNRFGSTNEIGI-FEMQSGGLVEVLNPSQVFLEERLDGATG------------ 290
                          :  :    * *                          :                .
RadA consensus                   KNRfG
```

c

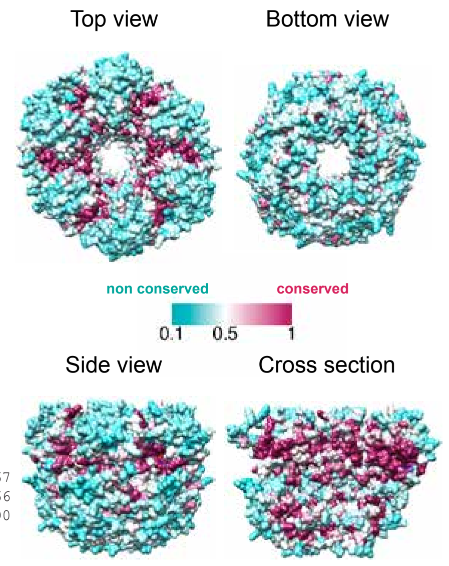

Supplementary Figure 7. Sequence analysis of RadA proteins.

(**a**) Distribution of RadA among prokaryotic and eukaryotic species. A non-exhaustive unrooted phylogenic tree of RadA is presented. It is based on the alignment of 140 protein sequences from annotated RadA genes picked randomly as representative of 24 bacterial phyla or from the Eukaryota domain on NCBI database (http://www.ncbi.nlm.nih.gov). RadA was absent from 2 bacterial phyla (Lentisphaerae and Tenericutes). The red arrow points to RadA from *S. pneumoniae.* All eukaryotic RadAs included in the tree share at least 40% identity with *S. pneumoniae* RadA and comprise the three domains C4, H, P (Fig. 1a). Sequence alignment was completed with Clustal Omega (http://www.ebi.ac.uk/Tools/msa/clustalo/) and the tree was constructed using the Neighbor Joining method and formatted with the iTOL online tool (http://itol.embl.de). (**b**) Sequence alignment of the helicase domains from *E. coli* DnaB and T7 Gp4 along with the H domain of *S. pneumoniae* RadA; "*", fully conserved residues; ":", residues from groups with very similar properties; "." residues from groups with weakly similar properties. The five SF4-motifs defining the H domains of the DnaB subfamily of SF4 helicases are highlighted in green; the consensus sequences for these motifs from 100 bacterial RadA proteins, together with the KNRFG motif, are shown in blue with strictly conserved residues in capital letters. (**c**) The conservation coloring profile as calculated by the ConSurf server (Landau et al., 2005) mapped onto the surface representation of the RadA structure. Conserved residues (maroon) cluster at the nucleotide-binding site, at the interface between the monomers in the hexamer and within the lumen of the R and P channels. Amino acids of average (white) and low (cyan) conservation are mostly located on the external surface of the ring, the dodecamerization interfaces and in the N-terminal loop.

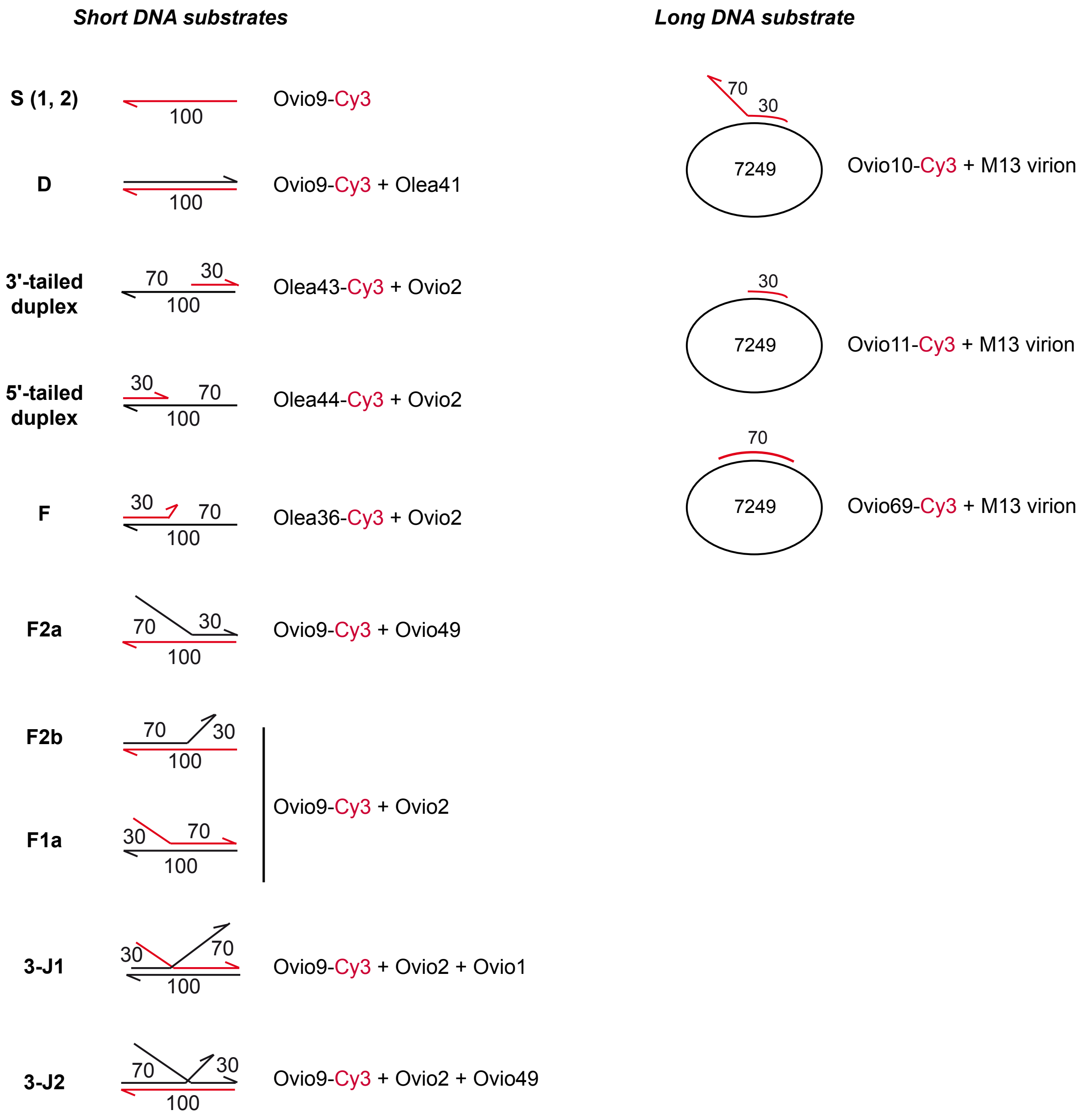

Supplementary Figure 8. DNA substrates for RadA biochemical analysis

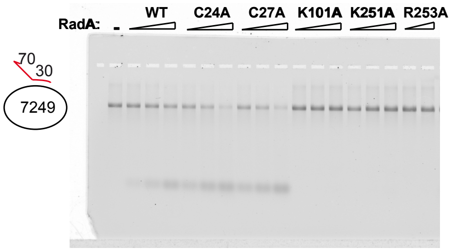

Gel related to Fig. 4c

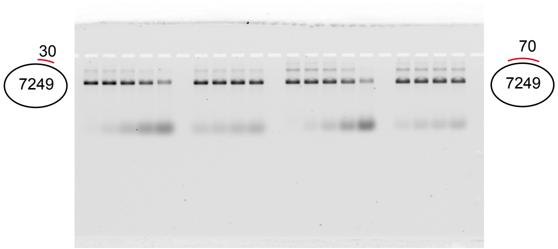

Gel related to Supplementary Fig. 4d

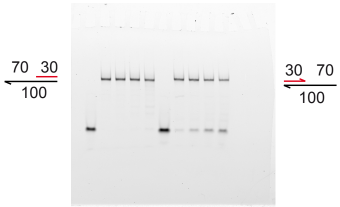

Gel related to Fig. 4e

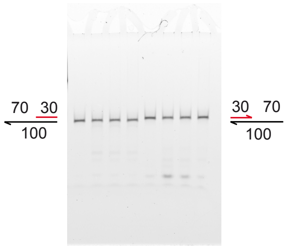

Gel related to Supplementary Fig. 6e

3-J1

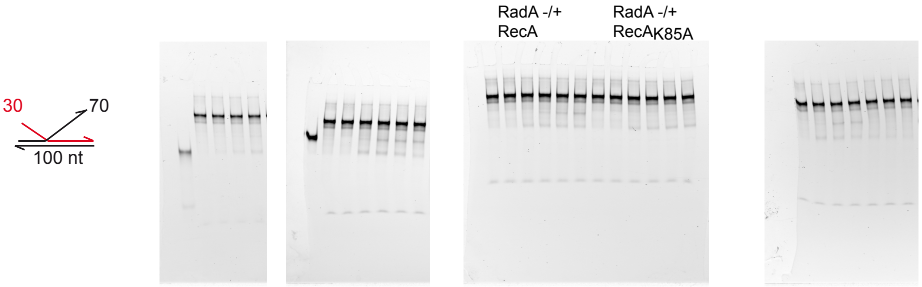

Gels related to: Fig. 5d Fig. 5f Fig. 5h Fig. 5i

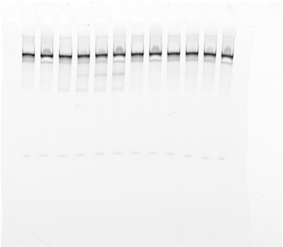

Supplementary Fig. 6f

3-J2

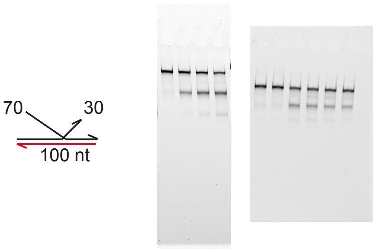

Gels related to: Fig. 5e Fig. 5f

Supplementary Figure 9. Raw gel images

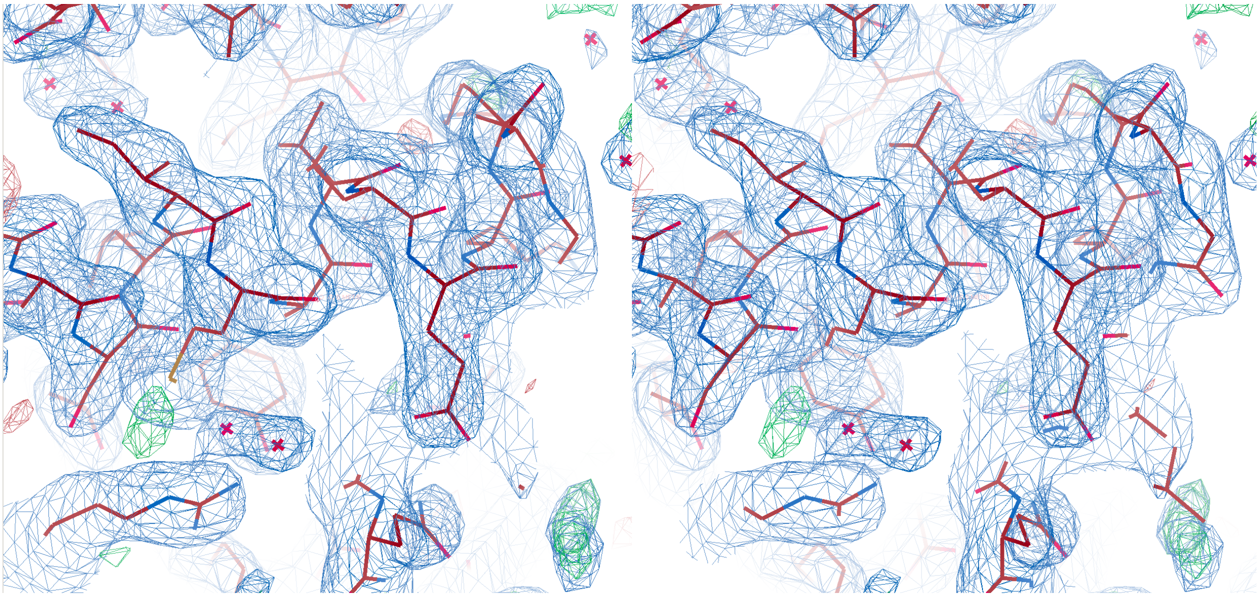

Supplementary Figure 10. Stereo View of 5LKQ

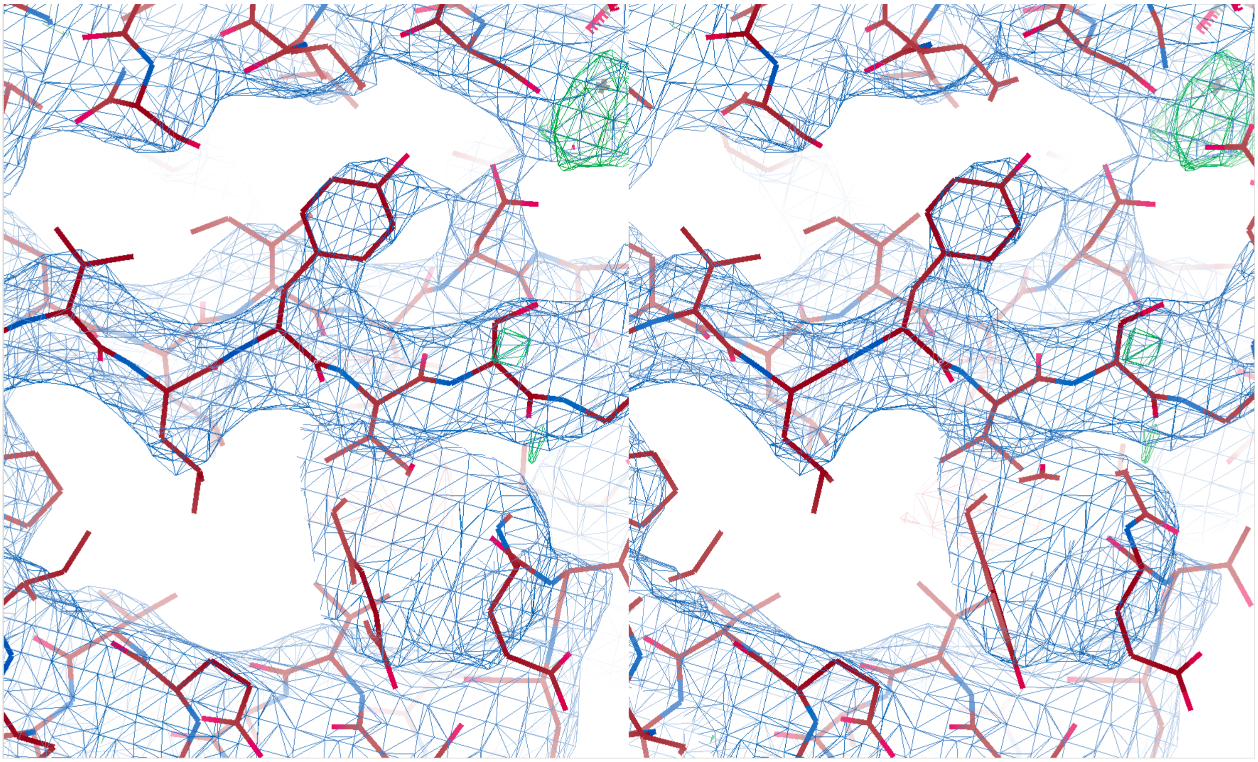

Supplementary Figure 11. Stereo view of 5LKM

Primer (nt)	Sequence (5' to 3')	Use in this work
MB117 (25)	AATCTCCGCTGTAGGTCACTTTCTT	*rpsL* PCRc (3434 pb)
MB120 (21)	TTGGATTGGGTGTGCATTTGC	
MB121 (23)	ACATGGACCTTCAGAGAAAGCCT	*rpsL* PCRe (4187 pb)
MB132 (26)	CCGCGTAAATCAAAAGTAGAAAAATC	
MB137 (23)	CGTCTAGGACACGCATGTCAAGA	*rpoB* PCRc (4195 pb)
MB138 (21)	GGCGGTAGACGGATTTGAACC	
MB139 (24)	TTCTTCTTGAATGGAGGGTTGAGC	*rpoB* PCRe (3912 pb)
MB140 (25)	GTCCGTGAACGTATGTCTGTTCAGG	
MB143 (23)	CGACCTACCAAATCAATCGGATT	*gyrB* PCRc (4114 pb)
MB144 (27)	GCCTTGAGAAAACGAAATGAATGGTTA	
MB145 (24)	GAAGAAAACAGGGTTCAACAACGG	*gyrB* PCRe (3896 pb)
MB146 (25)	GGGTCCGGTGTGAAGTGAACAGTTG	
OCN19 (49)	ATCTATGGCCCAGAGTCATCTGGTGCTACAACGGTTGCCCTTCATGCAG	$recA_{K85A}$ mutagenesis
Olea07 (37)	CCCCGGCCGTTATGCAAAGACCTTTTTCAAAACTTCC	*radA* cloning
Olea12 (56)	GGGAAGCTTGAAGGAGATTTGTCATGGCAAAGAAAAAAGCGACATTTGTATGTCAA	*radA* cloning
Olea13 (52)	GGTGGGGATCCTGGGATTGGGGCATCAACTCTTCTCCTACAAGTCTCAACCC	$radA_{K101A}$ mutagenesis
Olea15 (43)	CTGGGACGTTGCCCCAACGCTGGGTCTTGGTCTTCTTTTGTGG	$radA_{C27A}$ mutagenesis
Olea27 (51)	CGTATTTTGAGAGCGGTCAAAAATGCTTTTGGTTCCACTAATGAGATTGGG	$radA_{R253A}$ mutagenesis
Olea28 (50)	CCTTTCGTATTTTGAGAGCGGTCGCAAATCGTTTTGGTTCCACTAATGAG	$radA_{K251A}$ mutagenesis
Olea19 (23)	CCAAGTCAATCGCAGGTTCATCC	$radA_{C27A}$ SOEing PCR1
Olea15 (43)	CTGGGACGTTGCCCCAACGCTGGGTCTTGGTCTTCTTTTGTGG	
Olea20' (18)	GTTGGGGCAACGTCCCAG	$radA_{C27A}$ SOEing PCR2
Olea23 (23)	GCAGCCGACTGAGGTTCTCTACC	
Olea36-Cy3 (37)	**Cy3**-CTAGGGTCGGATCCTCTAGACAGCTCCATGTCCAGTG	Ovio2 partial complemental
Olea41 (100)	ACTTGATTCTGTCGCTACTGATTACGGTGCTGCTATCGATGGTTTAACGTCATAGACGATTACATTGCTATTCACACAGGAAACAGCTATGACCATGATT	Ovio9-Cy3 full complemental
Olea43-Cy3	**Cy3**-GCACCGTAATCAGTAGCGACAGAATCAAGT	Ovio2 partial

(30)		complemental
Olea44-Cy3 (30)	**Cy3**-CTAGGGTCGGATCCTCTAGACAGCTCCATG	Ovio2 partial complemental
Ovio1 (100)	CTAGGGTCGGATCCTCTAGACAGCTCCATGATCACTGGCAC TGGTAGAATTCGGCCCATTAGCAAGGCCGGAAACGTCACC CTCCAGTTTCTCGCCTCTG	Ovio2 partial complemental
Ovio2 (100)	ACTTGATTCTGTCGCTACTGATTACGGTGCTGCTATCGATG GTTTAACGTCATAGACGATTACATTGCTACATGGAGCTGTCT AGAGGATCCGACCCTAG	Ovio9-Cy3, Ovio1, Olea43-Cy3, Olea44-Cy3 partial complemental
Ovio8-Cy5 (100)	ACTTGATTCTGTCGCTACTGATTACGGTGCTGCTATCGATG GTTTAACGTCATAGACGATTACATTGCTAATTGTTATCCGCT CACAATTCCACACAACA-**Cy5**	Chase experiment
Ovio9-Cy3 (100)	AATCATGGTCATAGCTGTTTCCTGTGTGAATAGCAATGTAAT CGTCTATGACGTTAAACCATCGATAGCAGCACCGTAATCAG TAGCGACAGAATCAAGT-**Cy3**	Olea41, Ovio2, Ovio49 full or partial complemental
Ovio10-Cy3 (100)	AATCATGGTCATAGCTGTTTCCTGTGTGAAATCACTGGCAC TGGTAGAATTCGGCCCATTAGCAAGGCCGGAAACGTCACC CTCCAGTTTCTCGCCTCTG-**Cy3**	M13mp18 partial complemental
Ovio11-Cy3 (30)	**Cy3-**AATCATGGTCATAGCTGTTTCCTGTGTGAA	M13mp18 full complemental
Ovio49 (100)	CAGAGGCGAGAAACTGGAGGGTGACGTTTCCGGCCTTGCT AATGGGCCGAATTCTACCAGTGCCAGTGATTTCACACAGGA AACAGCTATGACCATGATT	Ovio9 partial complemental
Ovio69-Cy3 (70)	**Cy3-**TCCCAGTCACGACGTTGTAAAACGACGGCCAGTGCC AAGCTTGCATGCCTGCAGGTCGACTCTAGAGGAT	M13mp18 full complemental
**Strains**	**Genotype**	**Origin**
R1818	*comC0*, *hexA*Δ*3::ermAM*; Ery^{R}	*Caymaris et al., 2010*
R2194	R1818 but *radA::spcR (Burghout et al.,2007)*	*This study*

Supplementary Table 1. Oligonucleotides and strains used.
